# The Structure of the Chinese Material Value Scale: An Eastern Cultural View

**DOI:** 10.3389/fpsyg.2017.01852

**Published:** 2017-10-27

**Authors:** Jiangqun Liao, Lei Wang

**Affiliations:** ^1^Department of Psychology, Tsinghua University, Beijing, China; ^2^School of Psychological and Cognitive Sciences and Beijing Key Laboratory of Behaviour and Mental Health, Peking University, Beijing, China

**Keywords:** materialism value, happiness, success, possession, psychometric

## Abstract

This study investigated the structure of the Chinese Material Value Scale (MVS). A two-factor structure, rather than the original three-factor structure, was proposed for China by means of confirmatory factor analysis. Direct evidence showed that the dimensions of success and happiness could be merged together. Both explicit and implicit methods were used to examine the relationship between success and happiness based on possession. In particular, as an implicit method, the dot-probe paradigm recording participants’ response time supported the idea that the two-factors could be merged together. The results also showed that for Chinese people, success to an extent means happiness, while the converse is not necessarily true. Chinese are much more concerned about social evaluation than their own feelings, and this cultural characteristic is reflected in our findings.

## Introduction

Materialism fully developed during the industrial and post-industrial period (Belk, 1985). Materialism is the attachment of a high value to worldly possessions (Belk, 1984). Materialists place possessions and possession acquisition at the center of their lives, viewing them as essential to their satisfaction and well-being. They tend to judge their own and others’ success by the number and quality of their possessions (Richins and Dawson, 1992). Materialism is a psychological state that is closely related to people’s behaviors, which are of concern to scholars, social reviewers, and national public policymakers. Discretionary purchases and experiential consumption are important ways in which materialists gain psychological well-being (Mason, 1981; Dolan et al., 2007; Otero-Lopez et al., 2011; Richins, 2013; Brown et al., 2016; Pandelaere, 2016).

Culture has a strong influence on social cognition, behaviors, and values, and there are historical and cultural differences when it comes to materialism (Belk, 1984). Many studies have investigated the dimensions and level of material values in different cultures (see Richins, 2004). It is apparent that the structure of materialist values is shaped by cultural characteristics.

Ancient Chinese philosophers emphasized collective agency. Historically, Chinese value was greatly influenced by collectivist culture (Triandis, 1995). As described by Nisbett et al. (2001), Chinese are inclined toward holistic thinking; they were more likely to give “whole” response to a thing, in which all aspects of the thing are the basis of the response. This is one of the main characteristics of Eastern culture. Easterners tend to regard a larger number of factors as potentially relevant to explaining a given event. Chinese material values may also differ from those in individualistic cultures. Unlike individualistic cultures, which emphasize self-evaluation, collectivistic cultures emphasize social evaluation (Sun et al., 2014). As a social evaluation criterion, possession is commonly used to evaluate people’s success and happiness.

We contend that Chinese materialism will demonstrate a unique structure owing to its culture (collectivism). Chinese culture has affected people’s attitudes toward money, possession, success, and happiness for over a 1000 years (Bauer, 1976), which suggests a uniquely Chinese lifestyle different from that of Western culture. We therefore propose that the structure of materialism in China is different from that in Western countries. The present study was designed to investigate the dimensionality of materialism in Chinese samples to uncover culture-specific structural features.

## Conceptual Background

### Materialism and Material Value

Scholars have historically found that materialism is rooted in the West, where the modern industry and contemporary happiness-seeking lifestyle originated (Belk, 1985). It is consistently agreed that materialism is a product of economic development and market maturity. According to Engel’s coefficient, the per-capita GDP of $3,000 is an important economic development, signifying that the economy has reached the level of middle-income countries and that economic and social life in China will change. When referring to consumer behavior, people pay more attention to quality of life and consumption diversification.

Many researchers have defined and measured materialism from the perspective of attitude, personality, and values. For example, Campbell (1970) developed a materialism scale which focused on attitudes. Moschis and Churchill (1978) developed a materialism scale that included attitudes toward money as well as attitudes toward possessions. Focusing on personality traits, Belk (1985) developed a 24-item materialism scale with three-dimensions—envy, possessiveness, and non-generosity—a structure that seems inconsistent with the nature of materialism in less hedonic societies. Later, Ger and Belk (1990) added a fourth dimension: tangibility. Richins (1987) regarded materialism as an attitude and paid close attention to the meaning of possessions. Richins and Dawson (1992) further theorize that materialism is a value that influences the way people interpret their environment and constructs people’s lives. They define materialism as the importance attributed to possessions and the acquisition of material goods in achieving major life goals or desired states. They proposed an 18-item material value scale (MVS), which was shortened to 15 items by Richins (2004). Their MVS conceptualizes three-factors: the use of possessions to judge the success of others and oneself, the centrality of possessions in a person’s life, and the belief that possessions and their acquisition lead to happiness and life satisfaction. These factors are referred to as the success, centrality, and happiness domains, respectively.

As a value, material value reflects not only the natural attributes of wealth, such as the exchangeability of money, but also the social attributes of wealth, such as the social symbolism of possession. These features of material values are more easily influenced by culture. In the present study, Richins and Dawson’s (1992) MVS was chosen to be revised for several reasons. First, this scale reports report high internal reliability (Cronbach alphas for centrality, happiness, and success were 0.71–0.75, 0.73–0.83, and 0.74–0.78, respectively; for the combined scale varied between 0.80 and 0.88); Test–retest reliability (58 adults, 3-week interval) were 0.82, 0.86, and 0.82 for the three subscales, respectively, and 0.87 for the combined scale. Second, it relates to life values, and has been used in numerous studies in the US and elsewhere. Third, it is relatively short and easy to manipulate. Based on the literatures of MVS, Richins (2004) analyzed 15 raw date sets, reassess the MVS and revised the deficiencies of 18-item MVS. 15-item short version was proposed, which has better dimension properties than the 18-item version and the mean alpha was 0.86 for the combined 15-item one.

### Materialism Across Cultures

Materialism is affected by history, social economy, and culture (Belk, 1985). Family consumption and parenting style can foster materialism in the next generation (Richins and Chaplin, 2015). Kilbourne et al. (2005) found consistency in the structure of MVS among developed countries such as the United States, Canada, and Germany. For them, the most important factor was possession-defined success, followed by the pursuit of happiness and finally the centrality of acquisition. This research indicated that Richins and Dawson’s three-dimensional structure has consistently reflected Westerners’ material values, with each dimension clear and independent. Some scholars have studied the differences in Richins and Dawson’s material value between Eastern and Western cultures. For example, Webster and Beatty (1997) found that US and Thai consumers placed equivalent levels of importance on the centrality and happiness components, whereas Thai consumers tended to place more importance on the success component.

Ger and Belk (1990, 1996) showed that materialism is neither unique to the West nor directly related to affluence. This finding has been supported by other research (Feather, 1998). Among developed countries, for example, the level of materialism in American samples was higher than that in German samples; In the six countries (the Americans, New Zealand, Ukraine, Germany, Turkey, and Romania) they investigated, Romania, as a developing country, were found to be the most materialistic. Thai people, who are influenced by Eastern culture, also showed a higher materialism level than US. Tatzel (2002) reported that people in collectivist cultures such as East Asia are prone to being more materialistic than Westerners. Asians’ attitudes toward wealth reflect the public meaning of the self (Liao and Wang, 2009), which is a social attribute, whereas Westerners’ attitudes toward wealth reflect the private meaning of the self. People in East Asia often regard consuming capacity as a form of personal status (Wong and Ahuvia, 1998). When referring to material themes in magazine advertising, Japanese ads were found to be equally materialistic as US ads, but emphasized more status symbolism (Belk and Pollay, 1985a,b).

Clearly, there are differences between Easterners and Westerners on the dimensions of the MVS. These differences suggest that the structure of the MVS may also reveal cultural differences. However, the above studies did not examine the structural characteristics of the MVS in Eastern culture. Since materialism is affected by economy, culture, and history (Belk, 1985), it is necessary to study the structure of MVS in Eastern cultures such as China.

### Chinese Material Value

China is a society that emphasizes collectivism and personal social value (Hu, 1944; Ho, 1976; Hofstede, 1980; Krebs, 1982). Social evaluation is one of most important standards; people tend to feel success and happiness when they receive high levels of social evaluation. Possession, which can bring social status and reputation, is commonly used to evaluate success or happiness.

Chinese personal welfare, interest, and achievement are set collectively (Triandis, 1994); therefore, social status, roles, and relationships are explicit and public and play an important part in social life. In this context, social value is an important index of happiness and life satisfaction (Barger et al., 2009). Anything that can bring social status and reputation will bring happiness. Material success is one such thing. In conventional Chinese society, pursuing material success and surpassing others in life and career are the highest forms of success. The people with the highest wealth and status often represent the ultimate goal of happiness.

Economic conditions are the premise of materialism. In 2008, the Chinese per capita GDP entered the US $3,000 range for the first time (2009 China National Bureau of Statistics). The consumption of the Chinese people is characterized by diversification, and the materialistic values are becoming more and more mature (Sun et al., 2014).

Based the above review of the literature, we investigated the hypothesis that for Chinese people, the success domain and happiness domain of the MVS will be more closely related to each other, especially when the success and happiness can be represented through public possessions. Given the likely overlap between the two domains, we speculate that in China, the structure of material value may be two-dimensional instead of three-dimensional.

## Overview of the Present Research

We conducted two studies in mainland China. Study 1 examined the structure of Richins and Dawson’s (1992) MVS and its short version (Richins, 2004) in Chinese samples and revealed that there were only two-dimensions of materialism in China with the finding that the happiness factor and success factor merged together. In Study 2, we examined how strongly the items in the happiness and success domains of the MVS were associated with the concept of success and happiness through an implicit experiment, the dot-probe task. This further verified the overlapping of the items from the happiness and success subscales of the MVS.

The studies were approved by the Ethics Committee of Peking University and Tsinghua University. In Study 1, data were collected from different cities in Mainland, for example, Guangzhou, Beijing, and Shenzhen. Before completing the questionnaire, all participants read informed content and knew their rights. In Study 2, the participants were recruited from a public university. All participants gave written informed consent. The participants were informed that the questionnaire and the experiment did not involve any privacy. The participants could withdraw or terminate the experiment when they experience any discomfort during the experiment.

## Study 1: the Applicability of Richins and Dawson’S MVS in Chinese Samples

The aim of Study 1 was to examine the dimensionality of Richins and Dawson’s MVS in Chinese samples. We argue that Chinese people may have a unique structure of materialism, and the three-component structure of Richins and Dawson’s MVS may not fit a Chinese sample.

### Method

#### Participants

A total of 981 consumers from different cities in mainland China participated in the study. Participants included employees (*N* = 857), and students (*N* = 124). The mean age for the sample was 29.43 (*SD* = 7.73) and ranged from 18 to 78 years old. Among these participants, 452 were male, 68.4% had special training experience, and 32.2% had bachelor’s or higher degrees.

The participants were divided into two samples according to the number of questionnaire, namely, even-numbered sample (*N* = 490) and uneven-numbered sample (*N* = 491). One sample’s data was used for exploratory factor analysis, and the other sample’s data were used for confirmatory factor analysis (CFA).

In this study, the skewness coefficient of each item is between 0.04 and 0.72, and the absolute value of the Kurtosis is between 0.51 and 1.12. According to West et al. (1995), when the skewness and Kurtosis are less than 2 and 7, maximum likelihood estimation is robust. Thus, maximum likelihood estimation was used to handle missing data.

#### Measures

##### Material value scale

Material value scale (MVS) was developed by Richins and Dawson (1992). In 2004, Richins developed a 15-item short version.

In order to establish translation equivalence, Mullen’s (1995) method was used to translate the 18 original items of the Richins and Dawson’s (1992) scale into Chinese. A five-point Likert scale was used for all item ratings (1 = *strongly agree*, 5 = *strongly disagree*).

After all participants completed the MVS, the following scales were also tested to examine the validity of MVS. Of our participants, 375 completed the Oxford Happiness Questionnaire (OHQ) and Money Attitude Scale (MAS), and 145 completed the Money Ethic Scale (MES) and Social Comparison Scale.

##### Oxford happiness questionnaire

Psychological well-being was assessed with the OHQ (Hills and Argyle, 2002), devised as a broad measure of personal happiness. The Chinese version (Shi et al., 2005; Li and Chen, 2013) was used in the present study; the alpha coefficient was 0.90. Previous studies have revealed that individuals high on materialism have low psychological well-being (Richins and Dawson, 1992; Burroughs and Rindfleisch, 2002; Dittmar et al., 2014; Nagpaul and Pang, 2017).

##### Money attitude scale

The MAS was developed by Yamauchi and Templer (1982). The MAS has 29 items rated on a seven-point Likert scale ranging from “always” to “never.” This concept is indirectly related to materialism (Belk, 1985). The MAS has two subscales: Power–Prestige (measuring the degree to which one sees possession as a tool to influence and impress others) and Retention–Time (measuring the degree to which one plans for their financial future). We calculated both the total-scale and subscale scores to examine the criterion-related validity of the MAS. The alpha coefficient for total scale was 0.87; for the two subscales, the alpha coefficients were 0.89 and 0.82, respectively. The Chinese version (Hong et al., 2009) was used in the present study.

##### Money ethic scale (MES)

Tang (1993, 1995) developed the MES according to the ABC model of attitude toward money, which includes affective, behavioral, and cognitive components. The Chinese version (Wang, 2005) was used in the present study. We used the 15-item, five-factor (budget, evil, equity, success, and motivation) scale revised by Wang (2005), rated on a five-point Likert scale (Tang et al., 2003). The alpha coefficient for the total scale was 0.85. We predicted that the MES would be correlated with the MVS—the more people value money, the more inclined they are to pursue material wealth and define happiness and success by money, and the more they value material items.

##### Social comparison scale

Gibbons and Buunk (1999) developed the Social Comparison Scale, which includes 11 items. The Chinese version revised by Wang et al. (2006) was used in this study. People with high levels of social comparison are sensitive to the behavior of others, have a degree of uncertainty about themselves, and try their best to reduce self-uncertainty. They are interested in how others perform. The alpha coefficient for the total Social Comparison Scale was 0.85. with Westerners, East Asians differed in satisfaction with wealth regardless of social comparative experience (Ogden and Venkat, 2001). Status consumption is positively correlated to MVS and social comparison, and social comparison reflects the consumer behavior of pursuing status (Heaney et al., 2005).

#### Procedure

The MVS was distributed to the participants along with a consent form and demographic information sheet. Then other questionnaires for validity testing were completed. All participants were informed that completing the questionnaire was voluntary and anonymous.

### Results

#### Confirmatory Factor Analysis of the MVS

First, we examined Richins and Dawson’s (1992) MVS in Sample 1. The internal consistency (Cronbach’s alpha) was 0.80 for the 18-item scale, with 0.63, 0.63, and 0.54 for the three subscales, respectively; for the 15-item scale, Cronbach’s alpha was 0.79 for the overall scale and 0.62, 0.52, and 0.54 for the three subscales, respectively. Comparing with previous studies, the internal reliability of Richins and Dawson’s original MVS is better than this one. The results of the CFA of Richins and Dawson’s MVS, performed with Lisrel 8.80, revealed an unsatisfactory factor structure, with all absolute and incremental index values failing to meet the desirable levels. Specifically, the χ^2^ was highly significant (χ^2^ = 829.06, *df* = 132, *p* < 0.001), and the χ^2^/*df* value = 6.28, which was higher than the desired value of 3.0. (Wheaton, 1987). Moreover, the RMSEA was 0.103, indicating that the model of fit cannot be accepted; the GFI was 0.84. In addition, the three incremental fit indices (CFI = 0.79, NNFI/TLI = 0.75, RMSEA > 0.1) failed to reach the recommended criterion of 0.90. Additionally, the three subscales were highly correlated with each other (*r*_12_ = 0.97, *r*_13_ = 0.87, *r*_23_ = 0.84). We also conducted CFA for Richins’ (2004) short-version MVS. The results, similar to those of the long version, also revealed an unsatisfactory factor structure (see **Table 1**).

**Table 1:** Indicators of standardized CFA.

	Original MVS (*N* = 490, *p* < 0.001)					
Model	Items	χ^2^/*df*	GFI	CFI	NNFI	RMSEA (90%, CI)
Three-factor	18	948.19/132	0.83	0.76	0.75	0.103 (0.10, 0.11)
	15	491.93/87	0.88	0.84	0.80	0.10 (0.09, 0.10)

	**Revised MVS(*N* = 491, *p* < 0.001)**					
**Model**	**Items**	χ**^2^/*df***	**GFI**	**CFI**	**NNFI**	**RMSEA (90%, CI)**

Two-factor	18	553.05/134	0.89	0.87	0.85	0.08 (0.07, 0.09)
	15	325.22/89	0.92	0.91	0.90	0.07 (0.06, 0.08)

*χ^2^, normal theory weighted least squares chi-square; *df*, degrees of freedom; (χ^2^/df < 3); GFI, goodness-of-fit index (0.90 and above); CFI, comparative fit index (0.90 and above); NNFI, Non-Normed Fit Index (0.90 and above); RMSEA, root mean square error of approximation (RMSEA < 0.08).*

The results demonstrate that the Richins and Dawson’s (1992) MVS may have a different structure in Chinese samples than in American samples. The principal-component analysis was conducted with oblique rotation to identify the MVS dimensionality in Chinese samples. No restrictions were set limiting the number of factors in the analysis; thus, the items were free to load on any number of factors. The criterion for item inclusion was set with factor loadings > 0.30. Principal component analysis showed that there are two-factors whose eigenvalues were greater than one and those were factors were rotated by the method of Varimax with Kaiser Normalization.

The results of EFA yielded a two-factor solution with the 18 or 15 items grouping into two coherent and interpretable factors: (1) Happiness–Success (10 items in the 18-item version and 9 items in the 15-item version) and (2) Material Possession (8 items in the 18-item version and 6 items in the 15-item version). The two-factors accounted for 33.8% of the response variance in the 18-item version and 36.9% in the 15-item version.

The correlations of two subscale were low but statistically significant for both the 15-item (*r* = 0.28, *p* < 0.001, *df* = 488) and the 18-item inter-factor (*r* = 0.32, *p* < 0.001, *df* = 488). According to Devellis (1991), the subscales demonstrated acceptable levels of internal consistency (Cronbach’s alpha) with Happiness–Success (α = 0.80 for the 18-item and α = 0.79 for the 15-item) and Material Possession (α = 0.67 for the 18-item and α = 0.62 for the 15-item). For each version, all items loaded on the two-factors consistently and stably, without any double loading.

Compared with the three-factor model, the two-factor model was confirmed to be more suitable. This indicates that materialism values have a simpler structure in Chinese samples than in American samples. Specifically, the happiness and success domains converged as one-factor. To reexamine the new two-factor structure, we conducted a confirmative factor analysis using the data from Sample 2. The results showed that the two-factor model for both the long and short version, and the materialism value of the original and short-form questionnaire fit well (**Table 1**). Specially, the 15-item MVS had higher GFI (0.92), critical CFI (0.91) and NNFI (0.90), lower RMSEA (RMSEA = 0.07, 90% CI: 0.06–0.08) and inter-factor correlation (*r*_12_ = 0.45, *p* < 0.01). The results of CFA showed in **Table 1**. **Table 2** lists the results for the two-factor structure using exploratory factors analysis. Combine multiple indicators of EFA and CFA, such as factor loading, RMSEA, CFI, NNFI (TLI) etc., the structure model was acceptable (Baumgartner and Homburg, 1996). Briefly, EFA and CFA supported the hypothesis that the 15-item short-form MVS had a two-factor structure in Chinese samples. This indicates that the Chinese MVS is different from that of Westerners.

**Table 2:** Rotated component matrix^a^.

Items	Component	
	H&S	MP
(1) I’d be happier if I could afford to buy more things.	0.703	
(2) I like a lot of luxury in my life.	0.703	
(3) I like to own things that impress people.	0.693	
(4) Buying things gives me a lot of pleasure.	0.620	
(5) It sometimes bothers me quite a bit that I can’t afford to buy all the things I’d like.	0.611	
(6) I admire people who own expensive homes, cars, and clothes.	0.578	
(7) Some of the most important achievements in life include acquiring material possessions.	0.499	
(8) The things I own say a lot about how well I’m doing in life.	0.392	
(9) I wouldn’t be any happier if I owned nicer things.	0.359	
(10) The things I own aren’t all that important to me.		0.744
(11) I wouldn’t be any happier if I owned nicer things.		0.708
(12) I don’t place much emphasis on the amount of material objects people own as a sign of success.		0.637
(13) I put less emphasis on material things than most people I know.		0.581
(14) I try to keep my life simple, as far as possessions are concerned.		0.416
(15) I have all the things I really need to enjoy life.		0.348
Initial eigenvalues	3.86	1.67
% of variance	25.75	11.12

*Extraction method: principal component analysis. Rotation method: varimax with Kaiser normalization. ^a^Rotation converged in three iterations.*

Moreover, we investigated the reliability and validity of the two-factor Chinese MVS with 15 items. A total of 375 participants completed the OHQ and MAS, and 145 participants completed the MES and Social Comparison Scale.

#### Reliability

The alpha coefficient of the Chinese 15-item MVS was 0.81, and the alpha coefficient of the adjusted Spearman–Brown formula was 0.86. The alpha coefficient was 0.79 and 0.72 for the two-factors, respectively. After one and a half months, we randomly selected 52 participants to take the MVS again, and the test–retest coefficient was 0.87 (95%CI: 0.68–0.93, *p* < 0.01, *df* = 50).

#### Validity

##### Convergent validity

The Chinese MVS scores correlated with the OHQ scores (*r* = -0.13, *p* < 0.05, *df* = 373). The two subscale scores were also correlated with OHQ (H&S: *r* = -0.17, *p* < 0.05, *df =* 373; MP: *r* = -0.10, *p* < 0.05, *df* = 373, respectively), which was consistent with the results of Richins and Dawson’s (1992) study, providing evidence for the convergent validity of the scale.

##### Criterion-related validity

The Chinese MVS scores were significantly correlated with MAS scores (*r* = 0.32, *p* < 0.01, *df* = 373) and Power–Prestige scores (*r* = 0.45, *p* < 0.01, *df* = 373), providing evidence for the criterion-related validity of the scale. In particular, Subscale 1 (H&S) positively (though marginally) correlated with Retention–Time (*r* = 0.10, *p* = 0.057, *df* = 373), whereas Subscale 2 (MP) negatively correlated with Retention–Time (*r* = -0.11, *p* = 0.05, *df* = 373), showing that they are two different constructs. While Subscale 1 focuses more on current happiness, Subscale two represents long-term orientation. The Chinese MVS scores were also significantly correlated with MES scores (*r* = 0.43, *p* < 0.01, *df* = 143). Subscale 1 (H&S) was positively correlated with MES (*r* = 0.49, *p* < 0.01, *df* = 143), while Subscale 2 (MP) was insignificantly negatively correlated with MES. The Chinese MVS were also positively correlated with Social Comparison Scale scores (*r* = 0.38, *p* < 0.01, *df* = 143). These results showed that the two-factor structure Chinese version of the MVS had acceptable reliability and validity. The correlation coefficients and confidence interval of criterion validity were list in **Table 3** as following.

**Table 3:** The correlation between MVS and criterion validity.

	*N* = 375				*N* = 145	
	OHQ	MAS	Power–Prestige	Retention–Time	MES	SCS
MVS	Pearson *R*	–0.13^∗^	0.32^∗∗^	0.45^∗∗^	0.02	0.43^∗∗^	0.38^∗∗^
	95% CI upper	–0.22	0.22	0.38	–0.02	0.30	0.21
	95% CI lower	–0.01	0.40	0.52	0.19	0.51	0.48
MVS- H&S	Pearson *R*	–0.17^∗^	0.46^∗∗^	0.56^∗∗^	0.10^†^	0.49^∗∗^	0.33^∗∗^
	95% CI upper	–0.26	0.38	0.49	–0.22	0.38	0.20
	95% CI lower	–0.04	0.55	0.62	–0.01	0.57	0.44
MVS- MP	Pearson *R*	–0.10^∗^	0.02	0.11^∗^	–0.11^†^	0.06	0.20^∗^
	95% CI upper	–21	–0.09	0.01	–0.08	0.01	0.09
	95% CI lower	0.02	0.11	0.21	–0.13	0.13	0.31

*^∗^*p* < 0.05; ^∗∗^*p* < 0.01; ^†^*p* < 0.10.*

### Discussion

The results of Study 1 showed that happiness and success converged as one-factor in the Chinese sample while possession centrality remained an independent factor. This was inconsistent with previous findings in American studies that the MVS had three-factors, each with a clearly separate construction (e.g., Richins and Dawson, 1992; Richins, 2004). This inconsistency can be explained from three perspectives. First, the meanings of happiness and success and their subjective and objective expressions correlate to each other in some ways. Much research has indicated that the achievement of a successful career and personal goals is an important component of individual subjective well-being (e.g., Campbell et al., 1976; Lu and Shih, 1997; Furnham and Cheng, 2000a), that material possession plays an important role in happiness and success (e.g., Lu and Shih, 1997; Furnham and Cheng, 2000b), and that the individual’s achievement and material wealth exemplify not only happiness but also success (Argyle et al., 1989; Chiasson et al., 1996; Fan and Karnilowicz, 1997; Furnham and Cheng, 2000b; Lu, 2001).

With respect to materialism values, people enjoy material wealth as the foundation of happiness, and the objective expression of happiness is the foundation of psychological well-being. Moreover, the success that material wealth brings is an important component of an individual’s subjective well-being (Argyle et al., 1989; Chiasson et al., 1996; Fan and Karnilowicz, 1997; Furnham and Cheng, 2000a; Lu, 2001), objectively expressed as happiness. Therefore, the success and happiness that material wealth brings may relate to each other by both contributing to people’s psychological well-being.

The second explanation comes from the economical perspective. It has been shown that Teenagers growing up with low socioeconomic status are inclined to regard material wealth as their most important goal (Singhal and Misra, 1992; Kasser et al., 1995). In rapidly developing societies, people crave material wealth, especially in countries such as China that experience sharply accelerating urbanization and commercialization (Ger and Belk, 1996). This, to a certain extent, also promotes the development of Chinese materialism values and strengthens the idea that people use material wealth as one of the important standards of success and happiness.

Third, from a cultural perspective, Westerners regard their own happiness as an ultimate life goal. The individual is independent, having unique inherent characteristics—such as personality, ability, motive, and values—and his/her behaviors stem from the influence of these inherent characteristics (Sampson, 1988). Comparatively, East Asians consider whether their views or behaviors are in agreement with their in-groups or societies (Triandis et al., 1990); their happiness is influenced by external standards. For instance, societal approval, maintenance of public image, modesty, and respect of parents and elders are all considered to be important values. Therefore, material wealth that is apparent to the public is apt to become an important factor of Chinese life goals that connect success and happiness.

In sum, the meanings of happiness and success are similarly expressed in China. Based on scales measuring material values, happiness and success were not independent; instead, they merged together as one-factor. The question of how the two-dimensions merged remains, however. Do they simply overlap, or does one comprise the other? Does this phenomenon occur due to the specific items? These questions need to be examined by empirical studies.

## Study 2: Verifying the Dimension Mergence Via Implicit Study

In Study 1, we found that the Chinese MVS had only two-factors, with happiness centrality and success centrality converged into one-factor. However, Study 1 used an explicit self-report method. Some research indicates that self-report measurements can be influenced by social desirability (Himmelfarb and Lickteig, 1982; Paulhus, 1984; Schlenker and Weingold, 1989). In order to confirm whether Chinese people really think the concepts of happiness and success are similar, we conducted an implicit experiment. We adopted the dot-probe paradigm (Macleod et al., 1986) used to investigate attention toward emotions, such as anxiety symptomatology, depression, social threat, and positive words in attention bias studies (e.g., Macleod et al., 1986; Gotlib et al., 1988; Ruiz-Caballero and Bermudez, 1997; Bradley et al., 1998; Mogg and Bradley, 1999). Some studies have used this paradigm to test whether subjects show an attentional orientation toward or away from the target stimuli in research on social cognition, for example, with respect to race and crime (Eberhardt et al., 2004) or optimism (Segerstrom, 2001). By investigating the reaction time (RT) that each item in the dimension of success centrality and happiness centrality was perceived as success or happiness, we examined whether Chinese people would converge the success and happiness factors to one on an implicit cognitive level.

In a modified dot-probe diagram, we first presented each item (happiness centrality or success centrality) on a computer in each trial, and then presented a pair of category words together: happiness and success (the left–right order of the two words was arranged randomly). Based on attention bias theory, if participants focus on some meaning of one item (e.g., happiness), they would focus their attention on the position of the category word “happiness.” Therefore, the response time in which the probe stimulus (e.g., an arrow) in the same position was recognized and judged (e.g., for the direction of the arrow) would be shorter. If the participants focus on the meaning of an item in terms of both happiness and success, there is no attention bias toward the left or the right and no significant difference in RT. Based on this experimental paradigm, we examined whether and to what extent each item was implicitly associated with happiness and success. This allowed us to uncover how the success and happiness factors in the MVS relate to each other.

### Method

#### Participants

Thirty-two undergraduates enrolled at a large public university (14 men, 18 women) with an average age of 20.29 (*SD* = 1.51, range = 18–23 years) volunteered for Study 2. The participants who completed the experiment were paid for 10RMB. All participants were right-handed, had normal or corrected-to-normal visual acuity, and were naïve to the purpose of the experiment.

#### Apparatus and Materials

The modified probe detection task was programmed and presented using Presentation software (Schneider et al., 2002) at a 1,024 × 768 pixel screen. Participants were seated approximately 60 cm behind the computer screen.

The materials included priming sentences and category words. Ten priming sentences were taken from the two-dimensions of the Material Value Scale: happiness centrality and success centrality (e.g., own expensive cars, homes, and clothes). These sentences had been adapted to make all items appropriately equal or similar in length and in affirmativeness of the statement. Immediately after priming sentences, the category words “success” and “happiness” were presented in font size 30, 10 cm from the middle of the screen. Finally, a dot probe (up arrow or down arrow with 38 × 50 pixels) was presented, placed in the position of one of the category words. Participants were asked to respond by pressing either the up or down arrow button as quickly and accurately as possible, pressing the up arrow when the arrow on the screen pointed up and the down arrow when the arrow pointed down. Before each pair of category words and arrow was presented, a fixed cross (0.6 cm × 0.6 cm) appeared in the center of screen to attract the participant’s gaze.

In the pilot study, 16 students judged the consistency between each priming sentence and its original item in the MVS in a matching task. They were asked to draw a line between one of the 10 edited priming sentences (for example, own expensive homes, cars, and clothes.) in one column and one of the 10 original items (e.g., I admire people who own expensive homes, cars, and clothes.) from the MVS in another column if they believed the two had similar meanings. The results showed that, on average, participants correctly matched 90% of the items. We then revised the priming sentence according to the results to make sure the shortened sentence used for priming retained the same meaning as its original item in the MVS.

#### Design and Procedure

**FIGURE 1:**
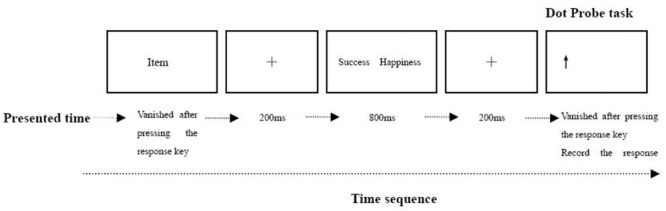
Time sequence of implicit experiment on a given trial. The display depicts the time sequence of a test session trial, which consisted of priming sentence, cross-fixed, two category words, cross-fixed and target probe. The actual stimuli were white against a black background.

Participants responded to six items during practice and 10 items in the formal experiment; each item was repeated 10 times. Priming sentences were presented by the computer one by one. The trials had no time limit; the sentence would not disappear from the screen until participants understood the meaning of the sentence and then pressed the space bar (see **Figure 1**). After refreshing, the screen showed a fixed cross (200 ms), followed by category words (800 ms), another fixed cross (200 ms), and an arrow, with the screen refreshing in-between. Each of the two category words (happiness and success) appeared on either the left or right side of the screen randomly; the arrow was placed in the position of any of the category words with equal probability and random order. The direction of the arrow was also random. Participants had to judge the direction of the arrow as quickly and accurately as possible. The entire experiment took around 15 min to complete.

We hypothesized that if participants thought the meaning of the item was success (happiness), their attention bias would be directed to the subsequent category word “success” (“happiness”), and the RT for judging the direction of the arrows would be faster when the arrow was presented in the position of the corresponding category word “success” (“happiness”) than that of “happiness” (“success”).

### Results and Discussion

The error rate, RT, Mean, *SD*, and results of the *t*-test are listed in **Table 4**. The RTs were analyzed using a 2 (arrow position: consistent/inconsistent) × 2 (category word: success/happiness) repeated measures analysis of variance. Both variables were within subject factors. Erroneous responses were discarded from statistical analyses, as were trials with RTs shorter than 300 ms or longer than 2 standard deviations above each participant’s mean RT.

**Table 4:** Response time results of implicit experiment.

		Happiness		Success	
		*Mean*	*SD*	*Mean*	*SD*
RT	*Arrow directed success*	494.57	58.34	492.37	56.10
	*Arrow directed happiness*	485.41	61.09	494.39	63.14
*t*		2.09^∗^		–0.42	
*df*		31		31	
*p*-Value		0.028		0.68	
Error rate		11.2%		11.8%	

*msec for RT. ^∗∗^*p* < 0.01; ^∗^*p* < 0.05.*

The results showed a non-significant two-way interaction between arrow position and category word (*F*[1,31] = 0.20, *p* > 0.05, η^2^= 0.01). There was a significant main effect of category word (*F*[1,31] = 4.57, *p* < 0.05, η^2^= 0.13), and *ns* main effect for arrow position (*F*[1,31] = 0.001, *p* > 0.05, η^2^= 0.001).

We further analyzed the difference using a *t*-test, which showed that, for the success factor items of the MVS, there was no effect in the judgment of arrow direction (*t*[31] = 0.42, *p* > 0.05, Cohen’ *d* = 0.11). Participants showed no significant difference in response between the arrow placed in the position of category word “happiness” (494.39) and the arrow placed in the position of category word “success” (492.37). In other words, no attention bias occurred. This result indicates that participants equally related the success factor items of the MVS to the concepts of success and happiness.

However, for the happiness factor items of the MVS, there was an effect in the judgment of arrow direction (*t*[31] = 2.09, *p* < 0.05, Cohen’ *d* = 0.53); there was a significant difference in participant response between the arrow placed in the position of category word “success” (494.57) and the arrow placed in the position of category word “happiness” (485.41). This result indicated that participants related the happiness items in the MVS more closely to the concept of happiness than to the concept of success.

These findings demonstrate that the Chinese views of happiness and success do relate to each other, confirming the result in Study 1 with implicit empirical evidence for convergence of material happiness and success. Moreover, this study shows that the relationship is not a simple overlaying between material happiness and success, but a comprising relationship. As the results show, material success is associated with the success and happiness concepts; that is, success is seen as indicating happiness, but happiness might not indicate success.

## General Discussion

The present research revealed that the MVS has a two-dimensional rather than three-dimensional structure in Chinese samples. Two of the original dimensions in Richins and Dawson’s MVS, happiness centrality and success centrality, converged as one-factor. These findings were confirmed by two studies using both self-report questionnaire and controlled experiment.

In societies that attach importance to social evaluation criteria, success, and happiness tend to be integrated into one-dimension, so that the three-dimensions of materialistic value are reduced to 2. Analysis indicated that the 15-item Chinese MVS has better psychometric properties than 18-item Chinese MVS, original MVS and original short version MVS. This 15-item scale showed acceptable levels of reliability and validity, and good fitness of CFA. We verified the finding by using both explicit and implicit methods. Our finding reveals that Chinese materialism has unique characteristics, and may help shape the theory of material value into a more nuanced, culturally sensitive one.

Richins and Dawson’s (1992) MVS has become one of the most widely used measurements of material value; it has shown good psychometric properties and has been validated in some cross-cultural studies. In the present study, the dimensionality and structure of the MVS were verified in China. We found no conflict with the previous material value theory developed by Richins and Dawson (1992). Moreover, we revealed new information that helps enrich the theory under the condition of social evaluation system. It is clear from our findings that cultural differences have an impact on the structure of materialism.

Moreover, the two-dimensional 15-item version has some advantages in application. First, the structure is clearer. It can explain the impact of culture on materialistic values. Secondly, it can save time and the capacity of the questionnaire. We call for more research to verfiy these advantages by comparing the two versions (original and short) in the future.

### Materialism under Social Evaluation

Our observations showed that material possession centrality remains an independent factor under social evaluation, whereas the happiness and success dimensions may converge together. This suggests that the attribution of possession is culturally independent, while attributions of happiness and success are shaped by culture.

In Study 1, happiness and success items appeared in the same dimension. For example, “I’d be happier if I could afford to buy more things” represented happiness in the original MVS, and “Some of the most important achievements in life include acquiring material possessions” represented success in the original MVS; in our version, however, they represent the same factor.

The convergence of the material happiness and success factors lies in cultural influence. Easterners emphasize social role and public perspective when managing the self and hold interdependent self-concepts, which is directly related to the core feature of materialism: public consumption and possession (Marcus and Kitayama, 1991, 1994; Richins, 1994). This might be the cultural root through which people relate material success with material happiness. Success can bring people high social status, good reputation, and great wealth (Ting-Toomey, 1988, 1998). In Eastern culture, materialism is still portrayed as attention to status (Belk, 1985); for example, a large number of China’s advertisements represent the symbolic meaning of status. Good social status is a reasonable result of success and an acceptable demonstration of happiness. Furthermore, Easterners hold a holistic thinking style, which leads to a halo effect when we evaluate others by means of overall impression. Therefore, people tend to believe that possession success means happiness.

### Methodological Implications for Future Research

We provided new methods to examine the structure of Richins and Dawson’s (1992) MVS in Chinese samples. Generally speaking, when measuring a concept using a scale, researchers have often used explicit Likert scales, which may be influenced by desirability and other biasing factors. This limits our understanding of the underlying logical relationships.

In the present research, we used both explicit and implicit experimental paradigms, which allowed us to double-check the overlapping relationship of factors in the MVS. This is particularly important in the research of values, since values are relatively socially desirable, and participants’ explicit response may be more likely to be influenced by other motivations rather than their real personal views.

### Limitation and Implications for Practice

This study explores the characteristics of material values of Chinese consumers from the perspective of culture. One contribution of this research is the recognition that consumers’ material values can be influenced by culture in economic life.

Several limitations are worthy of attention. The current study did not include samples from other countries, and there is also no comparison of cultural differences directly with other countries, the material values that explain multiculturalism need to be further validated. As the data are collected by the same method, common method variance should be considered. Due to the existence of social desirability, self-reported materialist values affected by social and culture cognition. Third, the cultural impact is very complicated. Future research can further explore the impact of culture on materialistic values by means of experimental approaches.

The results of the present studies have some implications for marketing. For example, China is shifting from a planned economy to a market economy. Chinese consumers are experiencing dramatic changes within the country’s economic system and consumer markets (Xiao, 1997). It is necessary to educate Chinese consumers on how to avoid economic, physical, and psychological damage and irrational behaviors caused by the market trap (Jin et al., 2015). Similarly, a developing market in a country with a specific culture should also consider local cultural and behavioral characteristics and adapt localized marketing strategies (Sun et al., 2014). Specifically, since Chinese people closely relate happiness and success, emphasizing face (Liao and Wang, 2009) and addressing how products can bring both happiness and success could result in better outcomes.

## Author Contributions

JL: conceived the research idea; made the research design; performed the studies; analyzed the data; interpreted the results; prepared manuscript; LW: conceived the research idea; organized the research; provided test materials and equipment.

## Conflict of Interest Statement

The authors declare that the research was conducted in the absence of any commercial or financial relationships that could be construed as a potential conflict of interest.
